# Myosin heavy chain 2A and α-Actin expression in human and murine skeletal muscles at feeding; particularly amino acids

**DOI:** 10.1186/1479-5876-10-238

**Published:** 2012-11-28

**Authors:** Britt-Marie Iresjö, Kent Lundholm

**Affiliations:** 1 Department of Surgery, Institute of Clinical Sciences, Sahlgrenska Academy, Sahlgrenska University Hospital, Gothenburg, Sweden

**Keywords:** Myosin, Actin, Amino acids, Skeletal muscles, Snat2

## Abstract

**Background:**

Protein dynamics during non-steady state conditions as feeding are complex. Such studies usually demand combinations of methods to give conclusive information, particularly on myofibrillar proteins with slow turnover. Therefore, time course transcript analyses were evaluated as possible means to monitor changes in myofibrillar biosynthesis in skeletal muscles in conditions with clinical nutrition; i.e. long term exposure of nutrients.

**Methods:**

Muscle tissue from overnight intravenously fed surgical patients were used as a model combined with muscle tissue from starved and refed mice as well as cultured L6 muscle cells. Transcripts of acta 1 (α-actin), mhc2A (myosin) and slc38 a2/Snat 2 (amino acid transporter) were quantified (qPCR) as markers of muscle protein dynamics.

**Results:**

Myosin heavy chain 2A transcripts decreased significantly in skeletal muscle tissue from overnight parenterally fed patients but did not change significantly in orally refed mice. Alpha-actin transcripts did not change significantly in muscle cells from fed patients, mice or cultured L6 cells during provision of AA. The AA transporter Snat 2 decreased in L6 cells refed by all AA and by various combinations of AA but did not change during feeding in muscle tissue from patients or mice.

**Conclusion:**

Our results confirm that muscle cells are sensitive to alterations in extracellular concentrations of AA for induction of protein synthesis and anabolism. However, transcripts of myofibrillar proteins and amino acid transporters showed complex alterations in response to feeding with provision of amino acids. Therefore, muscle tissue transcript levels of actin and myosin do not reflect protein accretion in skeletal muscles at feeding.

## Background

Several studies have reported on regulation of protein synthesis in skeletal muscles in fasted and fed state indicating considerably elevated synthesis during 2–3 hours postprandially [1-6]. Usually, such studies are based on estimates of protein synthesis by incorporation of labeled amino acids into newly synthesized proteins [7-10]; methods that are dependent on complex assumptions, related to distribution of tracers among intra- and extra cellular pools of amino acids [8,11,12], and represent expensive and complex analytical methods. [13,14]. Consequently, alternative methods are needed in clinical studies. Therefore, tracer independent methods, measuring initiation of translational phosphoprotein complexes as well as cellular alterations in transcript concentrations of regulatory and target proteins for synthesis should be of value from several perspectives.

Our previous studies have confirmed that extracellular provision of amino acids activates translation initiation of protein synthesis in skeletal muscle tissue during both oral and intravenous feeding [12,15,16]. Such induction of translation initiation may be triggered by concentration changes of amino acids outside or inside muscle cells through mTOR signaling without a critical presence of insulin or extracellular IGF-1 [17-19]. However, strictly controlled experiments, based on labeled amino acids, did not provide consistent results on amino acid stimulation of total muscle protein synthesis, due to tissue pool- and tracee uncertainties [12,20]. Therefore, the present study was conducted to evaluate how provision of extracellular amino acids influenced on cellular expressions and content of transcripts of amino acid transporters and myofibrillar MHC2A as well as α-actin as possible markers for the synthesis of contractile proteins in skeletal muscles at feeding relevant for clinical nutrition studies.

## Material and methods

### Patient studies

Twelve patients who underwent upper gastrointestinal tract surgery participated [15]. They were randomized to receive overnight constant infusions of either saline or TPN, (Kabiven Perifer) for at least 12 hours prior to surgery as described elsewhere [15]. All infusions continued until muscle biopsies were taken from the rectus abdominis muscle directly after induction of anesthesia. Muscle biopsies with remaining intact RNA from 10 out of 12 randomized patients were used in present analyses. Amino acid concentrations in blood and translation initiation factor analyses from study and control patients have been reported elsewhere [15].

### Animal experiments

Female, weight stable C57 BL/6 mice were used. They were either starved or refed with standard rodent chow (2016 Global Tekland®, Netherlands) and had always free access to water. Starved mice had no access to food overnight for 12 hours before termination, while refed animals were similarly starved overnight for 12 hours, but had then free access to food for 3 hours before termination [18]. Animals were killed by cervical dislocation and mixed hind limb muscles were excised and immediately frozen in liquid nitrogen. Muscle samples were stored at −70°C until RNA extractions were performed. All animal procedures were performed in accordance to national guidelines for animal research and approved by the regional animal research ethics committee in Gothenburg.

### Cell cultures

Rat myoblast L6 cells were seeded in 25 cm^2^ flasks, 48 well- or 6-well dishes and grown to confluence in Dulbecco’s modified Eagle’s medium, with 4.5 g/l glucose (DMEM), supplemented with 10% foetal bovine serum (FBS), 100 IU/ml penicillin, 100 μg/ml streptomycin and 2 mM L-Glutamine. At day 4, when cells were confluent, medium was changed to standard DMEM supplemented with 2% FBS. At day 5, medium was changed to DMEM with very low amounts of all amino acids (0,14 mM), and without addition of FBS and antibiotics. Cells were cultured for 24 hours and thereafter cells were given new medium with either 0.28 mM amino acids (referred to as low AA) or 9 mM amino acids in total (referred to as high AA), which equals the concentration in standard DMEM. Cells were then cultured from 0.5-18 hours before harvest. In some experiments only groups of amino acids were included at elevated (high) concentrations in the medium while the remaining amino acids were kept at very low amino acid concentration (0.14 mM). Cells used in array experiments were harvested after 18 hours of refeeding. Cells were kept in an incubator with 95% air, 5% CO_2_ environment during the entire experiment.

### RNA isolation and cDNA synthesis

RNA from L6 cells was extracted using RNeasy mini kit (Qiagen) with DNAse step included. Cells were lysed in RLT buffer according to kit instructions by adding lysis buffer directly to cells in the culture dishes. Cell lysates were collected and homogenized by flushing 10 times through a 20G needle. Skeletal muscle tissue was homogenized with an Ultra-Thurrax homogenizer and RNA from human and mouse muscle tissue was extracted by RNeasy fibrous tissue mini kit with DNAse step included. Total RNA concentrations were measured by spectrophotometer (Nanodrop ND-100) and RNA quality was checked using an Agilent 2100 bioanalyser and RNA 6000 Nano kit. One μg of total RNA was reverse transcribed to cDNA using oligo d(T)-primers according to kit instructions (Advantage® RT for PCR kit, Clontech). Positive and negative controls were included in each run of cDNA synthesis.

### Real-time PCR

Commercially available primers from Qiagen were used for analysis of human α-actin/ ACTA 1 (QT00199815), rat α-actin/acta 1 (QT01081374), human myosin heavy chain 2A/MYH2 (QT00082495), human SLC38A2/(Snat 2) (QT 00030499), rat Slc38a2/(Snat 2) (QT00186116) and mouse Slc38a2/(Snat2) (QT00129542). Real time PCR was performed using QuantiTect™SYBR®Green PCR kit (Qiagen) according to kit instructions. 2 μl of cDNA and 2 μl of premixed Quantitect primers were used for each reaction of 20 μl exept for rat acta 1 where 5 μl cDNA were used. For analysis of mouse α-actin/acta 1 (5′-3′ Forward; CCAGAGTCAGAGCAGCAGA, Reverse CACGATGGATGGGAACAC), mouse myosin heavy chain 2A (5′-3′ Forward; TGGAGGGTGAGGTAGAGAGTG, Reverse; TTGGATAGATTTGTGTTGGATTG) primers were used. PCR analysis was performed with the PerfeCTa SYBR Green SuperMix (Quanta Biosciences) with the following settings; 95°-10 sek, 60°-30 sek, 72°-30 sek. 2 μl of cDNA and 3 pmol of each primer were used to a reaction of 10 μl. Real-time PCR was performed on either a LightCycler 1.5 instrument or a LightCycler 480 (Roche). Quantitative results were produced by the relative standard curve method and results are given in arbitrary units. All samples were analyzed in duplicates and negative controls were included in each run. Results from human and mice experiments are related to the expression of GAPDH as housekeeping gene which did not change significantly at starvation - refeeding. Results from cultured cells are reported as expression/ 18S. Levels of 18S RNA expression are provided separately since neither GAPDH nor 18S levels were stable at all experimental conditions in cell culture experiments. Only acta 1 (α-actin) and Slc38a2 (Snat 2) transcripts were measured in cell culture experiments since Mhc 2A transcripts were below detection levels when analyzed by real-time PCR.

### Microarray experiments

Microarray analysis was performed on RNA from eight samples (4 starved, 4 refed). 500 ng of RNA from starved and refed cells (18 hour) were labeled with Cy-3-dCTP and Cy-5-dCTP respectively (Amersham BioSciences), in a cDNA synthesis reaction with Agilent Flourescent Direct Label. cDNA from starved versus refed cells were then hybridized in competition on Whole Rat Genome Microarrays (4x44K expression oligoarrays, Agilent) during 17 hrs followed by post-hybridization washes according to in situ instructions (Hybridization Kit Plus, Agilent). This provides relative changes in gene expression. Microarrays were quantified on Agilent G2565 AA microarray scanner and data were pre-processed in Feature Extraction 9.1.3.1 software program (Agilent). Dye-normalized, outlier- and background subtracted values were imported into GeneSpring GX 10 software program (Agilent) for further analysis.

### Subcellular fractionation and labeling of cells

**Figure 1:**
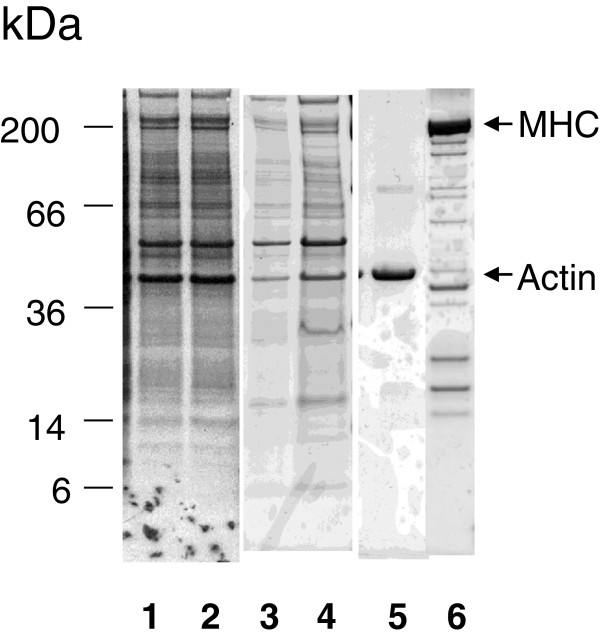
**Electrophoretic separation of cytoskeletal proteins in L6 cells by stepwise purification.** Cytoskeletal proteins were extracted using Calbiochems sub-cellular proteome extraction kit and separated by electrophoresis as described in Material and Methods. Lane 1–2 are autoradiograms of ^35^S-methionine labeled cells from low AA (lane 1) and high AA refed (lane 2) cells. Lane 3–4 are the same samples stained by Coomassie brilliant blue. Lane 5 is purified rabbit muscle actin (Sigma-Aldrich A-2522). Lane 6 is purified rabbit muscle myosin (Sigma-Aldrich M-1636). It has been demonstrated that L6 cells contain myosin [44].

Subcellular separation of the L6 cells was performed to check for protein expression of cytoskeletal proteins. L6 cells contained both actin as well as myosin heavy chains (Figure 1). Cells were labeled with ^35^S-Methionine as described elsewhere [12]. Both specific radioactivity and tracee concentrations of methionine were held constant in cell cultures when grown in presence of high or low amino acid concentrations. Stepwise subcellular fractionation of cells was performed by using Proteoextract® Subcellular proteome extraction kit (Calbiochem, Merck Biosciences) according to kit instructions. Cell fraction 4, which contains cytoskeletal proteins, was separated by electrophoresis and proteins were either stained by Coomassie brilliant blue or visualized by autoradiography as described elsewhere [12].

Incorporation of L-[U-^14^C]-phenylalanine (40 μCi/μmol phe) to cellular proteins was performed as described elsewhere in the presence of low or high medium concentrations of amino acids in the presence of constant concentration of phenylalanine (12μM).

#### Statistics

Results are presented as mean ±SE. Group comparisons were performed by factorial ANOVA followed by Fisher PLSD post hoc testing. p<0.05 was regarded statistically significant in two-tailed tests. Statistics used in the array experiment are described in the results section.

## Results

### Changes in transcript levels of myofibrillar proteins

**Figure 2:**
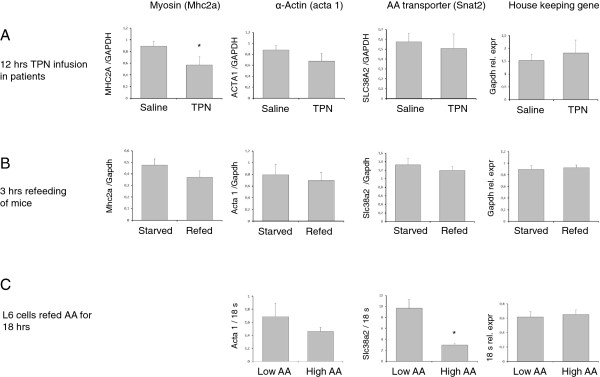
**mRNA levels of Myosin heavy chain 2A, ACTA 1 and SLC38A2 in three different models with increased protein synthesis and translation initiation at amino acid provision.** Rectus abdominus skeletal muscle tissue from surgical patients who received total parenteral nutrition compared to saline infusions for 12 hours; Mice “refed” with standard chow for 3 hours after 12 hours of starvation and L6 skeletal muscle cells cultured in presence of either low (0,28mM) or high (9 mM) total amino acid concentrations for 18 hours. Muscle MHC2A transcript levels were significantly decreased in parenterally fed patients (n=5) compared to saline infused control patients (n=5) (* p<0.05) while Mhc 2A /acta 1 transcrips were not different in mixed hind limb muscle tissue from starved/refed mice (Acta 1 p<0.10, MCH2A p<0.18, n=16) or cultured L6 cells (p<0.3). Slc38a2 (Snat2) levels were significantly decreased in L6 cells cultured in presence of high amino acid concentrations (p<0.01).

Skeletal muscle tissue from surgical patients, who received 12 hours continuous infusion of total parenteral nutrition, displayed significantly decreased MHC2A transcript levels compared to muscle tissue from control patients receiving saline only (p<0.05), while ACTA 1 transcripts were numerically decreased but did not reach statistical significance (Figure 2A). Similarly, both Mhc2A and acta 1 transcripts appeared to decline in skeletal muscles from refed mice compared to starved mice, but the difference did not reach statistical significance (Mhc2a p<0.18, Acta 1 p<0.10, n=16; Figure 2B). Similar findings occurred for Acta 1in confluent L6 cells refed low (0.28 mM) vs. high AA (9 mM) concentrations during 18 hours ( Acta 1, p<0.3, n=14; Figure 2C).

**Figure 3:**
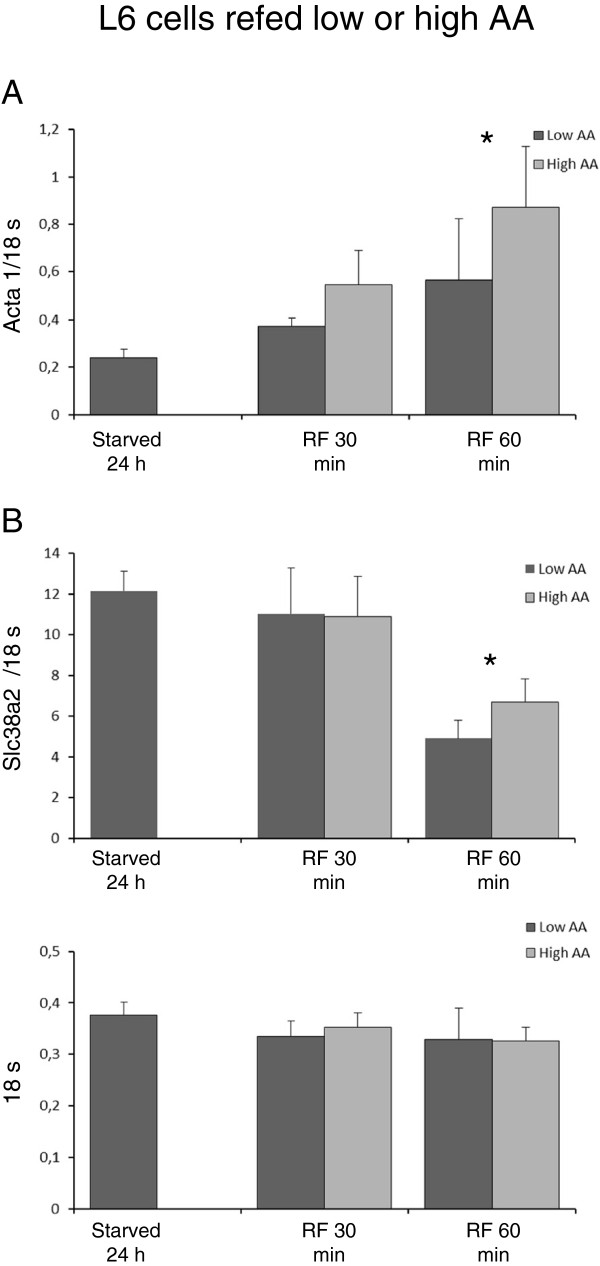
**mRNA levels of acta 1 and slc38a2 in confluent L6 cells refed low (0.28 mM) or high (9 mM) total amino amino acid concentrations compared to cells cultured for 24 hours in starvation medium (0,14 mM) as described in Materials and methods.** Acta 1 was significantly increased in low and high AA refed cells at 60 min compared to 24 hours starved cells (p<0.01). Slc38a2 concentrations were decreased at 60 min (p<0.05, n=9).

**Figure 4:**
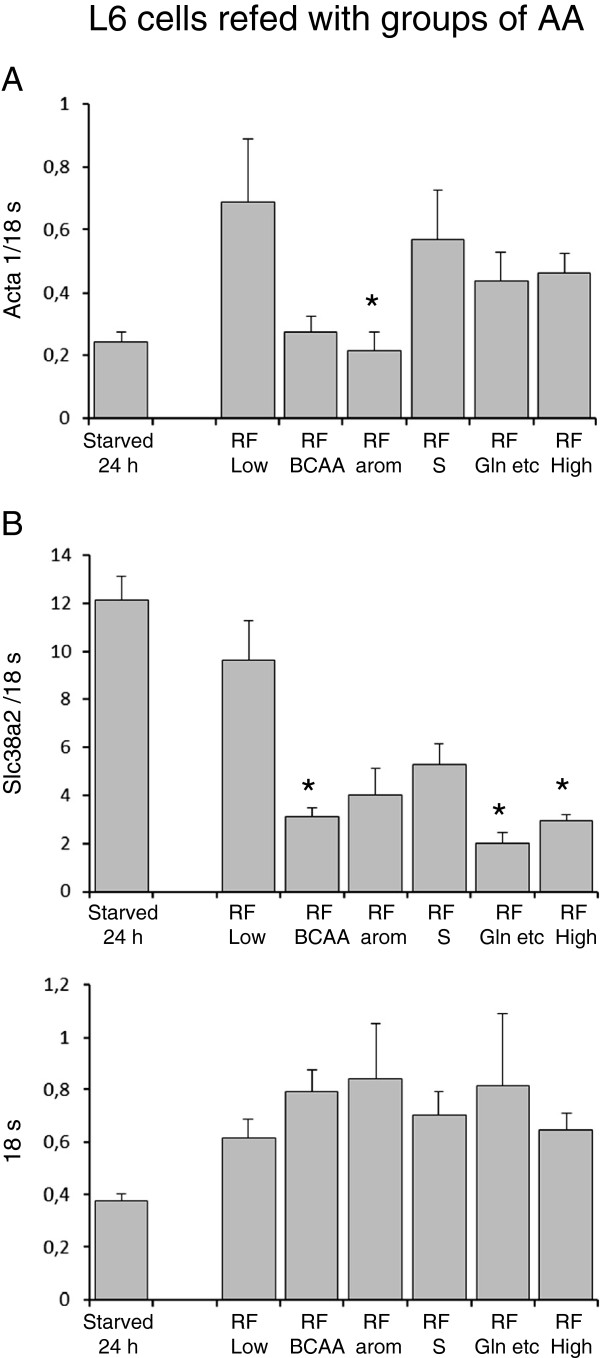
**Transcript levels of acta 1and slc38a2 (Snat 2) in confluent L6 cells refed various groups of amino acids.** L6 cells were cultured in the presence of low AA (0.28 mM) or high AA (9 mM) concentrations of all amino acids, or in the presence of high concentrations of various groups of amino acids (BCAA, aromatics, gln) in addition to background concentration of all amino acids in DMEM (0.14 mM) as described in Material and Methods. All cells were cultured in medium with decreased amounts of all amino acids (0.14 mM) for an initial period of 24 hours. Media were changed and cells were cultured in the presence of either low AA (0.28 mM), high AA (9 mM) or group amino acids for further 18 hours. (* p< 0.05 vs. refed low). RFBCAA = refed by BCAA (2.5 mM), RFArom = refed by aromatic amino acids (1mM), RFGlu = refed by Gln, Lys, Arg, Thr, His (5.3 mM).

Acta 1 transcripts increased significantly at 60 min of AA refeeding compared to 24 hours starved cells (cultured in presence of very low AA concentrations (0.14 mM) for 24 hours, before start of refeeding) with no difference between low and high AA groups (Figure 3A; n=9). Acta 1 levels remained increased at 4 and 8 hours compared to 24 hours starved cells, without any differences between low and high AA groups (4 hours; Low AA 0.818±0.273, High AA 1.141±0.796 , 8 hours; Low AA 1.152±0.740, High AA 0,992±0,330; n=3). Refed confluent L6 cells, based on different groups of AA at high concentrations for 18 hours, did not result in clear-cut significant alterations of Acta 1 levels among the groups (Figure 4A).

### Changes in transcript levels of amino acid transporter slc38a2/Snat 2

SLC38A2 (Snat2) levels did not differ between skeletal muscles from TPN and saline infused patients (P<0,7) as well as in refed mice compared to starved mice (Figure 2A,B). In contrast, Slc38a2 (Snat2) levels were significantly lower in L6 cells refed high AA concentrations during 18 hours compared to cells receiving low amino acid concentrations. (p<0,001) (Figure 2C). The discrepancies between slc38a2 transcripts at 60 min (Figure 3B) and 18 hours (Figure 4B) appeared to emerge beyond 4 hours incubation at high versus low AA concentrations (4 hours; Low AA 14.7±3.2, High AA 10.4±2.5, 8hours; Low AA 19,6±5.1, High AA 5.0±1.3; n=3) Based on these findings we chose 18 hours incubation for comparisons among groups of AA experiments. Cellular Slc38a2 (Snat2) transcripts were thus influenced by refeeding by various combinations of amino acids for 18 hours. Cells refed by branched chain amino acids (leu, ile, val) or by glutamine in the presence of other non-essential amino acids (Arg, Thr, His, Lys) showed decreased levels of Slc38a2 (Snat2) transcripts, while refeeding by aromatics (phe, tyr, trp) or sulphur amino acids (met, cys) in the presence of all amino acids in DMEM at low background concentration (0.14 mM) did not alter Slc38a2 mRNA levels (Figure 4 B).

### Total protein synthesis in cultured L6 cells

**Figure 5:**
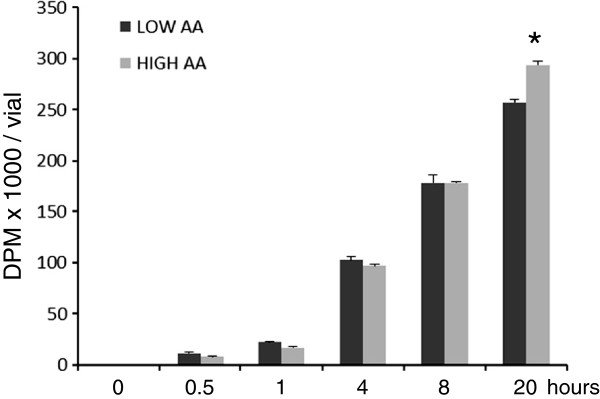
**Time course changes in L-[U-**^**14**^**C]-phenylalanine incorporation into proteins.** Confluent L6 cells were cultured in medium containing very low amounts of amino acids (0,14 mM) for 24 hours, and then refed in low (0,28 mM) or high (9 mM) concentration of total AA concentrations, with constant specific radioactivity of phenylalanine (40 μCi/μmol phe). Cells were harvested by TCA-precipitation of proteins. (* p< 0.01 vs. low AA).

Incorporation of ^14^C-phenylalanine into total cellular proteins increased continuously in the presence of amino acids and was significantly higher in the presence of high amino acids in the medium after 20 hours incubation compared to cells incubated with low amino acid concentrations (Figure 5).

### Microarray results

Of 41 090 probes on the Agilent 4 × 44K whole genome rat array, 22 318 entities remained after filtering of flags to remove low expressed genes. Statistical evaluation by *t*-test with Benjamini-Hochberg correction for multiple significance showed that 6675 entities were significantly different between starved and refed cells (p< 0.05). 745 entities showed at least 2 fold changes and were used for further analysis (399 up-, 346 down-regulated). A search by name (actin, myosin, tropo and slc) among the significantly changed transcripts with a fold change above 2 was performed in order to find mRNAs for amino acid transporters and contractile proteins. Several amino acid transporters demonstrated alterations in expression levels in response to provision of amino acids to cell cultures (Table 1). A gene ontology (GO) analysis was performed to find categories/pathways with significant enrichment of genes. Ten GO categories were found; all related to lipid, cholesterol and steroid metabolism (Table 2). Differentially expressed entities in GO cathegories “steroid biosynthethic and metabolic processes” are presented in Table 3. These results provide evidence that amino acid have profound effects on intermediary and nitrogen metabolism in muscle cells.

**Table 1:** Altered transcript levels of amino acid transporters and muscle proteins in confluent L6 muscle cells refed high amino acid concentrations versus low amino acid concentration assessed in microarray experiments as described in Material and Methods

**Agilent entity**	**Foldchange array**	**Foldchange Q-PCR**	**Common name**	**Gene symbol**	**Protein name/Function**
A_44_P463878	**−3.2**	**−2.6**	NM_181090	Slc38a2	Snat 2/ System A transporter
A_44_P393273	**−3.0**		NM_053818	Slc6a9	Glyt1/ System Gly transporter
A_44_P104652	**−2.7**		NM_181090	slc38a2	Snat 2/ System A transport
A_44_P510515	**−2.3**		NM_017206	Slc6a6	Taut/ System BETA, taurine transport
A_44_P410954	**−2.0**		ENSRNOT00000011006	Slc43a1_predicted	Lat 3/ System L-like transporter
A_44_P994686	**−3.6**		NM_012676	Tnnt2	Troponin T2, cardiac
A_42_P786933	**−2.4**		NM_012983	Myo1d	Myosin 1D
A_44_P489468	**−2.1**		ENSRNOT00000030661	ENSRNOT00000030661	Myosin heavy chain, smooth muscle isoform

**Table 2:** GO categories with significant enrichment of entities in L6 cells refed high amino acid concentrations

**GO ID**	**GO Accession nr**	**GO Term**	**Corrected p-value**
8636	GO:0016126	sterol biosynthetic process	1.289 E-12
4323	GO:0006695	cholesterol biosynthetic process	3.812 E-9
4322	GO:0006694	steroid biosynthetic process	9.406 E-8
8635	GO:0016125	sterol metabolic process	9.406 E-8
5578	GO:0008610	lipid biosynthetic process	7.391 E-7
4260	GO:0006629	lipid metabolic process	4.741 E-6
18769	GO:0044255	cellular lipid metabolic process	3.867 E-6
3797	GO:0006066	alcohol metabolic process	2.552 E-6
5262	GO:0008203	cholesterol metabolic process	2.552 E-6
5261	GO:0008202	steroid metabolic process	1.768 E-4

Based on entities selected by fold change ≥2.

**Table 3:** Altered transcript levels (entities) in GO categories “steroid biosynthetic and metabolic processes” in confluent L6 muscle cells refed high amino acid concentrations

**Agilent entity**	**Foldchange array**	**Foldchange Q-PCR**	**Common name**	**Symbol**	**Name**
A_44_P365580	**6.0**		NM_017136	Sqle	Squalene epoxidase
A_42_P814765	**5.8**		NM_001013071	Tm7sf2	Transmembrane 7 superfamily member 2
A_43_P16774	**5.6**	**6.5**	NM_001006995	Acat2	Acetyl-Coenzyme A acetyltransferase 2
A_42_P794613	**5.1**	**6.5**	NM_031062	Mvd	Mevalonate (diphospho) decarboxylase
A_44_P251944	**5.0**		NM_053539	Idi1	Isopentenyl-diphosphate delta isomerase
A_43_P12843	**4.5**		NM_053539	Idi1	Isopentenyl-diphosphate delta isomerase ]
A_44_P512136	**4.5**		NM_022389	Dhcr7	7-dehydrocholesterol reductase
A_44_P487240	**3.6**		NM_017268	Hmgcs1	3-hydroxy-3-methylglutaryl-Coenzyme A synthase 1
A_44_P168285	**3.5**		NM_012941	Cyp51	Cytochrome P450, subfamily 51
A_43_P11890	**3.4**		NM_017268	Hmgcs1	3-hydroxy-3-methylglutaryl-Coenzyme A synthase 1
A_44_P422696	**3.2**	**4.1**	NM_031541	Scarb1	Scavenger receptor class B, member 1
A_42_P796502	**3.1**		NM_017235	Hsd17b7	Hydroxysteroid (17-beta) dehydrogenase 7
A_44_P334218	**3.0**		NM_031049	Lss	Lanosterol synthase (Lss)
A_44_P379244	**2.9**		NM_031541	Scarb1	Scavenger receptor class B, member 1
A_44_P432965	**2.8**		NM_031840	Fdps	Farensyl diphosphate synthase (Fdps)
A_43_P22542	**2.7**		NM_001009399	Nsdhl	NAD(P) dependent steroid dehydrogenase-like
A_44_P347250	**2.6**		NM_001080148	Dhcr24	24-dehydrocholesterol reductase
A_43_P13043	**2.6**		NM_057137	Ebp	Phenylalkylamine Ca2+ antagonist (emopamil) binding protein
A_44_P143567	**2.5**		NM_031840	Fdps	Farensyl diphosphate synthase
A_44_P315661	**2.4**		NM_013134	Hmgcr	3-hydroxy-3-methylglutaryl-Coenzyme A reductase
A_43_P13088	**2.2**		NM_080886	Sc4mol	Sterol-C4-methyl oxidase-like
A_43_P11729	**2.0**		NM_013134	Hmgcr	3-hydroxy-3-methylglutaryl-Coenzyme A reductase
A_44_P237994	**−2.1**		NM_053502	Abcg1	ATP-binding cassette, sub-family G (WHITE), member 1
A_44_P536613	**−2.1**		NM_001025415	Ch25h	Cholesterol 25-hydroxylase

## Discussion

A large number of studies have evaluated rates and translation initiation of total protein synthesis in skeletal muscles in response to feeding during recent decades [13,21]. However, such studies, mainly based on incorporation of labeled amino acids, suffer from uncertainties and complex assumptions for calculation of protein synthesis rate [10,11,22-24]. Difficulties occur particularly at rapid alterations in bio-dynamics during non-steady state conditions [10,14]. Therefore, alternative and tracer independent methods have recently been applied in both animal and clinical experiments, complementary to tracer based methods. Such techniques are mainly based on assessment of phosphorylation/de-phosphorylation of regulatory proteins or protein complexes related to translation initiation of proteins where advantages are straight forward assessment of protein phosphorylation status in cells and tissues under evaluation without the need of steady state [6,15]. Tissue sampling and processing are comparatively easy and analytical principles are robust at standardized conditions [15]. However, limitations are that results reflect only initiation of overall protein bio-synthesis and do not reflect alterations of defined proteins. Determinations of the amount of a particular protein(s) in skeletal muscle tissue should in part resolve this problem, but is only applicable in long-term experiments, since it would be practically difficult to correctly assess quantitative alterations of defined proteins in skeletal muscle cells during short-term responses. Therefore, it should be possible to obtain relevant information by assessment of tissue transcript levels of defined myofibrillar proteins in response to feeding as applied in studies on orally refed healthy volunteers [25]. Unexpectedly, it was then observed that oral refeeding caused a decline of myofibrillar transcripts in skeletal muscles, at conditions otherwise associated with anabolic metabolism [16,26-28]. Such transcript information was seen in the light of observations that stimulation of gene transcription is usually reflected by increased tissue levels of transcripts for defined proteins aimed at subsequent translation to meet cellular requirements. [29-32]. Therefore, expected findings should be that net efflux of amino acids from skeletal muscles, due to increased net protein breakdown, should be associated with postprandial down-regulation in transcription of myofibrillar proteins. Normal oral feeding which is leading to rapid and pronounced activation of skeletal muscle protein synthesis, should then be characterized by increased transcription of required proteins [33]. Based on this simplistic view, we decided to re-evaluate effects on transcripts of myofibrillar proteins as Myosin heavy chain 2A (myosin) and acta 1 (α-actin) in skeletal muscle tissue in response to refeeding, particularly with focus on effects by amino acids in both patient and animal experiments.

Myosin heavy chains contributes to 20-25% of overall muscle protein synthesis in humans [34,35] while actin may display both lower and higher turnover compared to mixed muscle proteins [35,36]. Muscle tissue is however composed of many different proteins where sarcoplasmatic and myofibrillar proteins have different basal turnover and synthesis rates at feeding [37]. Adult human muscle tissue expresses three different isoforms of myosin heavy chain (MHC-I, MHC-IIa and MHC-IIx), where MHC-IIa is highly expressed in humans, while rodents express one additional form (MHC-IIb) [38].The myosin gene family is located in a cluster on chromosome 17 in humans and on chromosome 11 in mice [39]. Studies have indicated that mRNA content of different myosin isoforms correlates to the relative content of various MHC proteins present in skeletal muscle tissue [40,41] Changes in expression patterns of myosin heavy chain proteins exist in skeletal muscles during hypertrophy in the control of net muscle mass subsequent to loading [42-46], but less is known in response to feeding, although Mhc 2X mRNA is reported to unexpectedly increase after 7 days at reduced oral intake in rats [47]. Our present findings show that that transcripts of myosin heavy chain 2A and actin appeared to decrease during continuous TPN administration in agreement with previous findings showing decreased MHC 2X mRNA levels at 3 hours after oral meal intake [25];conditions that provide increased formation of eIF4G·eIF4E complex and decreased association of 4E-BP1·eIF4E [15].

There may be several reasons why myosin transcripts do not clear-cut reflect transcriptional activities and translational needs in cells during continuous long term nutrition exposure, although Rennie and coworkers [48] have reported transient changes in myofibrillar protein synthesis suggesting that muscle cells become refractory to amino acids in response to oral bolus feeding. However, long term provision of intravenous nutrition to patients leads to both time-proportional increases in muscle mass and continuously increased incorporation of labeled amino acids during the presence of high amino acid provision as seen in our present cell experiment (Figure 5). Therefore, it appears that transcript cellular levels of actin and myosin are influenced by a variety of factors that possibly determine absolute levels in both short and long term perspectives at nutrition.

It has never been finally assessed how amino acids signal across cell membranes to elicit triggers for induction of translation initiation, although it is assumed there are extracellular/intracellular amino acid sensors since muscle cells are sensitive to alterations of amino acid concentrations [49]. Recently, amino acid transporter proteins gained increased interest based on their ability to sense amino acid changes and influence intracellular signaling [50]. Regulation of expression of amino acid transporters may thus be an important part of the cell machinery in control of protein synthesis secondary to amino acids availability [49,51]. Therefore, we investigated how transcription of the transporter protein Snat 2 (encoded by the gene slc38a2) was affected by refeeding in our models. Snat 2 is a transporter of neutral amino acids belonging to system A [52]. Several amino acids in the refeeding medium (glutamine, histidine, cysteine, methonine) are transported by Snat 2, while branched chain- and aromatics are transported by system L across cell membranes [51,52]. Amino acid transporting by system A increased following amino acid deprivation [53]. Accordingly, we found that Snat 2 mRNA was lower in refed L6 cells compared to starved cells, although such alterations were not evident in vivo. Concentrations of Snat 2 mRNA were also decreased in refed cells by a group of amino acids (Gln, His, Lys, Arg, Thr). Refeeding L6 cells by branched chain amino acids decreased Snat 2 mRNA, although transported by system L, which operates by 1:1 amino acid exchange, which may couple influx of branched chain amino acids to efflux of cytoplasmatic amino acids such as glutamine [54]. It is possible that refeeding cells with branched chain amino acids caused either efflux or influx of other amino acids, which may alter Snat 2 mRNA levels. If so, Snat 2 should be influenced by extra cellular concentrations or transmembrane fluxes of either Gln or His, since it was not changed by refeeding of cysteine or methione which are Snat 2 substrates.

Our microarray data on cultured cells confirm that amino acids have pronounced effects on steroid and lipid metabolism in skeletal muscle cells. Only GO categories/pathways related to lipid and steroid metabolism showed significant enrichment, although microarray experiments indicated that a large number of individual transcripts (30%) were changed following amino acid provision. It has been reported earlier that skeletal muscle cells are capable of local synthesis of sex steroid hormones [55], and there are several ways for cells to provide cholesterol for use in steroid synthesis, such as the mevalonate pathway, where cholesterol is synthesized through a series of enzyme reactions from Acetyl CoA and HMG-CoA [56,57]. Thus, it was interesting to find that transcripts of all enzymes in this pathway were increased following amino acid provision to L6 cells. The expression of steroids and enzymes increases after exercise and may therefore represent an important part of anabolism following physical training in skeletal muscles [58]. Thus, results in the present study confirm that amino acids have profound metabolic effects upstream to initiation of protein synthesis in cultured isolated skeletal muscle cells, as observed in animal and human skeletal muscle tissue [12,59-64], in part related to individual groups of amino acids [65-69], as also observed in human biopsy specimens [17,61].

## Conclusion

In conclusion, previous and present studies confirm that skeletal muscle cells are sensitive to alterations in extracellular concentrations of amino acids for translation initiation of protein synthesis, usually indicated by polysome aggregation, increased incorporation of amino acids into cellular proteins and activation of translation initiation [12,15,19]. However, transcripts of myofibrillar proteins and amino acid transporters showed unexpected complex time course changes in response to various conditions of refeeding and should therefore be used only in combination with other indicators of muscle protein synthesis. Thus, tissue levels of actin and myosin transcripts are not suitable as in vivo markers for protein accretion in skeletal muscles in response to feeding.

## Competing interests

The authors declare that they have no competing interests.

## Authors’ contributions

BI conducted all experiments and statistical analyses. KL conceived of the study. Both authors participated in design of the study, drafted the manuscript and approved the final version.
